# Evaluation of Tumor Treatment of Magnetic Nanoparticles Driven by Extremely Low Frequency Magnetic Field

**DOI:** 10.1038/srep46287

**Published:** 2017-04-11

**Authors:** Weitao Li, Yangyang Liu, Zhiyu Qian, Yamin Yang

**Affiliations:** 1 Department of Biomedical Engineering, Nanjing University of Aeronautics and Astronautics, 29 Yudao Street, Nanjing, 210016, China

## Abstract

Recently, magnetic nanoparticles (MNPs), which can be manipulated in the magnetic field, have received much attention in tumor therapy. Extremely low frequency magnetic field (ELMF) system can initiate MNPs vibrating and the movement of MNPs inside of cells can be controlled by adjusting the frequency and intensity of ELMF towards irreversible cell damages. In this study, we investigated the detrimental effects on tumor cells with MNPs under various ELMF exposure conditions. An in-house built ELMF system was developed and utilized for evaluating the treatment efficiency of MNPs on tumor cells with specific intensities (2–20 Hz) and frequencies (0.1–20 mT). Significant morphological changes were found in tumor cells treated with MNPs in combing with ELMF, which were consistent with noticeable decrease in cell viability. With the increase of the intensity and frequency of the magnetic field, the structural integrity of tumor tissue can be further destroyed. Destructive effects of MNPs and ELMF on tumor tissues were further determined by the pathophysiological changes observed *in vivo* in animal study. Taken together, the combination of MNPs and ELMF had a great potential as an innovative treatment approach for tumor intervention.

Owing to their superior physiochemical properties, nanoparticles have been applied in various biomedical fields as a promising and viable technology, with intense researches focused on their potential applications in tumor intervention^1^,^2^,^3^,^4^,^5^. Due to their small size in nanoscale, nanoparticles can pass through various physiological barriers and/or penetrate into cells^6^,^7^,^8^. Among them, magnetic nanoparticles (MNPs) consisting of elements such as iron, nickel and cobalt exhibit unique physical features that make them a useful tool for both medical diagnostics and therapy. In particular, as MNPs can be manipulated by an external magnetic field^9^,^10^,^11^,^12^, their interaction with cells can be confined to targeted area to avoid any unwanted side effects. MNPs have been widely utilized in drug delivery^13^,^14^,^15^, hyperthermia treatment for cancer^16^, magnetic targeting^17^, contrast agents in magnetic resonance imaging (MRI)^18^, cell labeling and sorting^19^,^20^, and immunoassays^21^. Although extensive studies have demonstrated the relative biological safety of MNPs^22^,^23^,^24^,^25^, prior to build effective MNPs systems for various biomedical applications, their cytotoxicity and critical characteristics including size, structure, surface functionality, magnetic properties, stability, and dispensability^26^,^27^ have to be taken into full investigation.

Considering the magnetic properties of MNPs, different electric and magnetic fields can be applied toward various biomedical purposes. For example, when applying for hyperthermia treatment^28^,^29^,^30^, MNPs suspension has a high efficiency to absorb the energy of an altering magnetic field and convert it into heat. However, high concentrations of MNPs and magnetic field with high frequency and intensity are often required for this approach to generate sufficient heat that can kill cells, which would result in unwanted side effects. Alternatively, noticed the advantages of extremely low frequency magnetic field (ELMF), the investigations of the properties of ELMF, including intensity, frequency and time windows have been attracted much attention.

Previous studies have shown that damages on cell membranes could be generated by electric impulses or periodic electric field^23^. By tuning the pulse time or the electric intensity, micropores formation on cell membrane and structure changes of cells could be permanent towards irreversible cell damage. Similarly, under the exposure of external ELMF, MNPs could vibrate locally and the speed and moving direction of MNPs could be adjusted by the properties of ELMF. Therefore, with the assistance MNPs inside of tumor cells, it is thus assumed that the loss of membrane integrity and morphological changes could be achieved under ELMF exposure, which could consequently result in tumor cell damage.

In this study, we developed a novel ELMF system and evaluated the treatment efficiency on tumor cells with MNPs in ELMF with specific intensities and frequencies. Both *in vitro* and *in vivo* experiments were performed to determine the cell morphological changes and cell destruction following the exposure of ELMF with the presence of MNPs. To the best of our knowledge, no research has been explored in investigating the damage effects of MNPs and ELMF on tumor cells, which demonstrate promising potentials for tumor treatment.

## Results

### Characteristics of MNP-Fe_3_O_4_

**Figure 1:**
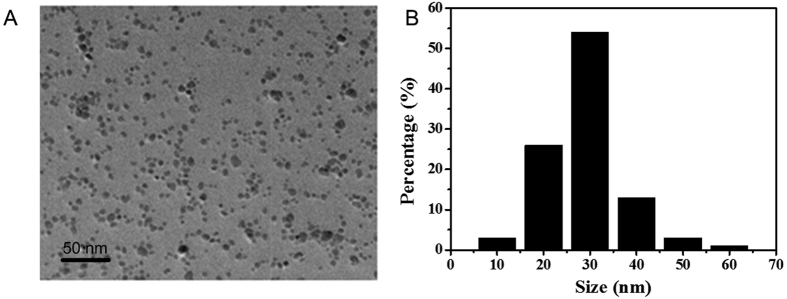
Characteristics of MNP-Fe_3_O_4_. (**A**) TEM images of MNP-Fe_3_O_4_. (**B**) Histogram of the distribution of hydrodynamic size of MNP-Fe_3_O_4_ obtained by DLS.

MNP-Fe_3_O_4_ coated with meso-2,3-dimercaptosuccinic acid (DMSA) was applied in present study. As shown in Fig. 1A, transmission electron microscopy (TEM) images of MNP-Fe_3_O_4_ showed sphere-like structures and were well-distributed. From dynamic light scattering (DLS) analysis results (Fig. 1B), the average hydrodynamic size of MNP-Fe_3_O_4_ was 30 nm with the average kernel diameter at 7 nm, suggesting that the particles were relatively homogeneous and well suspended. Surface modification of DMSA on MNP-Fe_3_O_4_ could maintain the stability of nanoparticles in biological solutions and also provide good biocompatibility.

### Cell viability in the presence of MNP-Fe_3_O_4_ without magnetic field exposure

**Figure 2:**
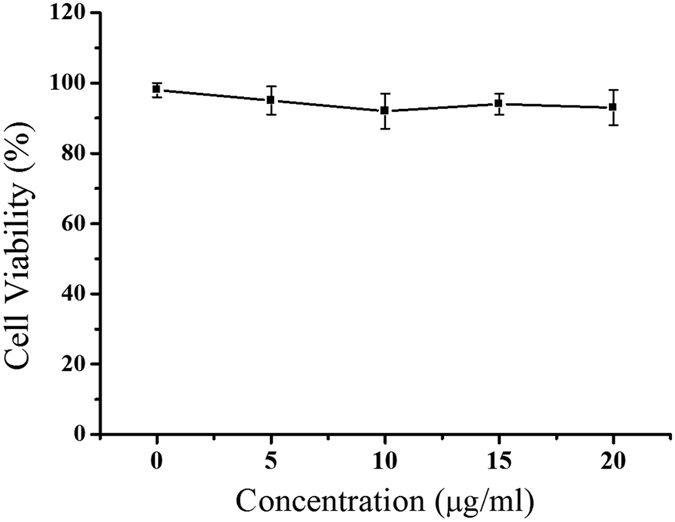
The cell viability results of MCF-7 cells treated with MNP-Fe_3_O_4_ at different concentrations of [0, 5, 10, 15, 20] μg/ml without any magnetic field exposure. Culture without any treatment was used as control. The cell viability value was quantified by MTT assay, and results are expressed as the percentage of viable cells relative to untreated control.

The cytotoxicity of MNP-Fe_3_O_4_ at different concentrations was obtained from cells treated without any magnetic field exposure. Cell viability is presented as the percentage of viable cells treated with MNP-Fe_3_O_4_ relative to the untreated control. As shown in Fig. 2, without external magnetic field, MNP-Fe_3_O_4_ showed negligible cytotoxicity to MCF-7 cells, with cell viabilities above 90% at all applied MNP-Fe_3_O_4_ concentrations.

### Morphological changes of cells exposed to ELMF

**Figure 3:**
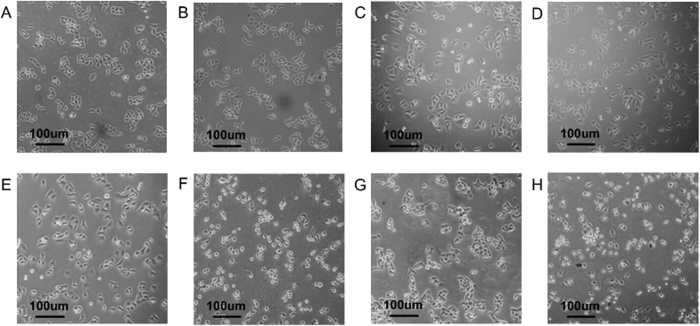
Bright field images of cells after different ELMF treatment. Top row: representative images of MCF-7 cells without MNPs incubation, exposed with ELMF at (**A**) 1 mT and 2 Hz; (**B**) 1 mT and 20 Hz; (**C**) 10 mT and 2 Hz; (**D**) 10 mT and 20 Hz; Bottom row: representative images of MCF-7 cells incubated with MNPs (10 μg/ml) and exposed with ELMF at (**E**) 1 mT and 2 Hz; (**F**) 1 mT and 20 Hz; (**G**) 10 mT and 2 Hz; (**H**) 10 mT and 20 Hz. Scale bar: 100 μm.

To study the effects of ELMF on cells, in our study, an innovative ELMF system was developed to generate alternating magnetic field with specific intensity and frequency. As shown from Fig. 3A to Fig. 3D, in the groups exposed to different ELMF but without the presence of MNPs, it was found that majority of cells retained their normal polygonal shape with clear sharp boundaries between cells. As MCF-7 cells intend to grow in an aggregated manner and form clusters in monolayer culture, we can observe the typical cell aggregation in them. However, with the addition of MNPs (bottom row in Fig. 3), significant morphological changes of cells were observed in accordance with the increasing intensity and frequency of external ELMF. Cells loaded with MNPs and exposed with ELMF became round and showed obvious abnormal morphology. In addition, cell-cell interaction was crucially damaged as we can observe the detachment of the cells, with diminished cell aggregation. With the increasing intensity and frequency of ELMF, damages on cells became much more severe, with significant smaller cell size and lower cell density found in the group exposed to ELMF of 10 mT at 20 Hz (Fig. 3H).

### Cell viability test upon ELMF exposure

**Figure 4:**
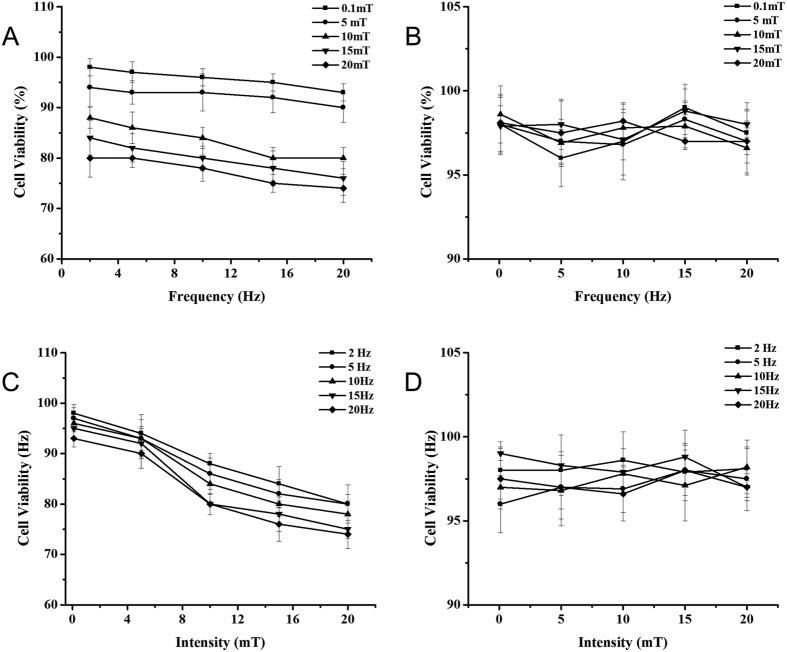
The cell viability results of MCF-7 cells incubated with MNPs (10 μg/ml) and exposed with ELMF. Frequency dependence of cell viability in cells treated (**A**) with and (**B**) without MNPs and exposed with ELMF at intensity of [0.1, 5, 10, 15, 20 mT]. Intensity dependence of cell viability in cells treated (**C**) with and (**D**) without MNPs and exposed with ELMF at frequency of [2, 5, 10, 15, 20] Hz. Cells without any treatment was used as control in each group. The cell viability value was quantified by MTT assay, and results were expressed as the percentage of viable cells relative to untreated control.

In addition to the morphological observation of structural changes in cells, cell viabilities after ELMF exposure under various conditions were analyzed quantitatively. As shown in Fig. 4, without the addition of MNPs, ELMF by itself did not cause any remarkable changes on cell viability. Despite the difference in the frequency and intensity of ELMF, cell viabilities remained stable (above 95%) and comparable to negative control group without any MNPs or ELMF treatment. In contrast, with the presence of MNPs in cells (Fig. 4A and C), significant descending trends in cell viability can be observed with the increase of magnetic field intensity and frequency.

**Figure 5:**
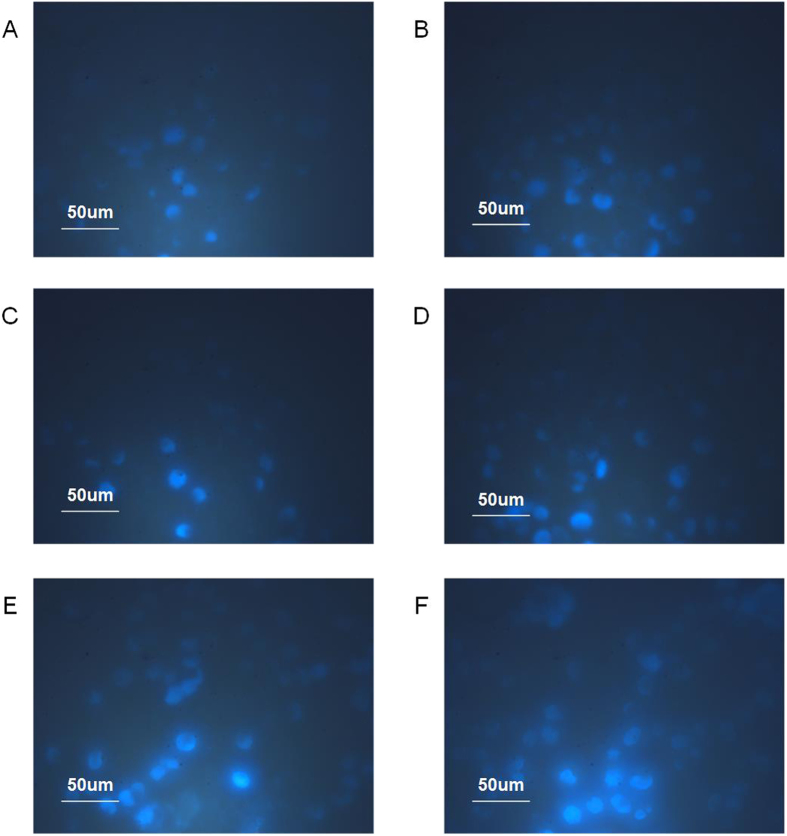
Representative fluorescence images of cells followed by different treatments. Cells were stained with apoptotic cell Hoechst 33258 dye kit. (**A**) cells treated without MNPs but with ELMF exposure; (**B**) cells incubated with MNPs (10 μg/ml) but without ELMF exposure; cells incubated with MNPs (10 μg/ml) and exposed to ELMF at (**C**) 1 mT and 2 Hz; (**D**) 1 mT and 20 Hz; (**E**) 10 mT and 2 Hz; (**F**) 10 mT and 20 Hz. Scale bar: 50 μm.

To further determine the cytotoxic effects of ELMF exposure in combining with MNPs on tumor cells, apoptotic cell Hoechst 33258 dye kit was employed to stain the cells after various treatments. As the loss of cell membrane integrity and highly condensed chromosomes normally can be found in cells undergoing apoptosis, Hoechst 33258 dye will be uptaken more easily and show more significant fluorescence signals in apoptotic cells as compared to healthy cells. As shown in Fig. 5, the presence of MNPs in cells under ELMF exposure obviously generated more significant fluorescent signals, indicating distinct apoptotic process could be triggered following the destruction of the cell membrane integrity. With the enhancement of the magnetic field intensity, fluorescence signal in cells also increased, suggesting that the apoptotic effects on cells are dependent on the intensity of ELMF.

### Effects of ELMF exposure combining with MNPs in animal models

**Figure 6:**
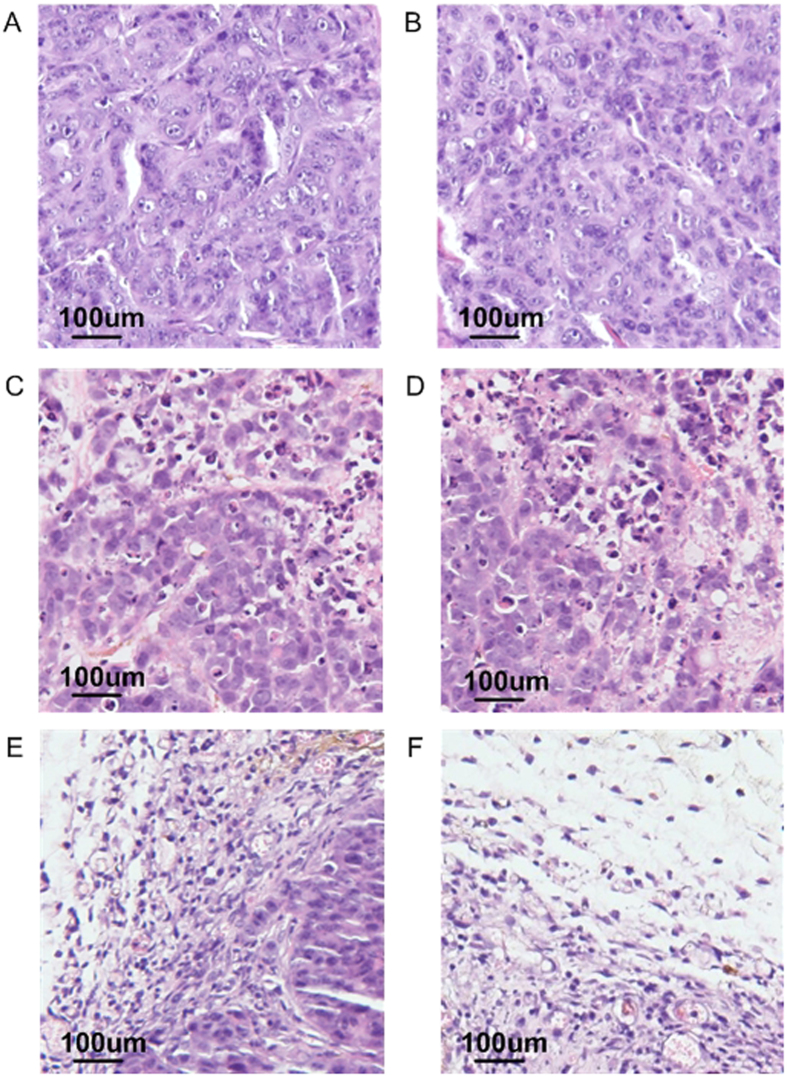
Representative histology images of tumor tissues followed by different treatments. Mice injected intratumorally with (**A**) physiological saline but with ELMF exposure; (**B**) MNPs (10 μg/ml) but without ELMF exposure. Mice injected intratumorally with MNPs (10 μg/ml) and exposed to ELMF at (**C**) 1 mT and 2 Hz; (**D**) 1 mT and 20 Hz; (**E**) 10 mT and 2 Hz; (**F**) 10 mT and 20 Hz. Tumors from mice treated with MNPs and ELMF showed distinctive characteristics of cellular damage, such as abundant pyknosis, karyorrhexis, and karyolysis. Scale bar: 100 μm. Thin cross-sections were stained with H&E.

Followed by intratumoral injection of MNPs and ELMF exposure, tumor tissues from mice were collected and processed for fixation. The cross-section of tumor tissues was histological stained with hematoxylin and eosin to further visualize and assess cell death. In the mice treated without MNPs but with ELMF exposure (Fig. 6A) and in the group incubated with MNPs but without ELMF exposure (Fig. 6B), tumor tissues remained in an intact and compact format, with healthy nuclei and complete cytoplasms in cells. In contrast, with the combining effects of MNPs and ELMF, significant changes were evident in accordance with intensity and frequency of the applied ELMF. In general, MNPs-loaded tumor cells after ELMF treatment were apparently destroyed, which appeared abnormal structure with necrotic changes such as fragmented nuclei. When comparing the effects of magnetic field of same intensity yet at different frequency, it was found that higher frequency may result in more significant cell damages (Fig. 6C vs. 6C, and Fig. 6E vs. 6F). With the increase of the intensity of the magnetic field, the structural integrity of tumor tissue was further destroyed, remaining the matrix and debris of damaged cells (Fig. 6E and F).

## Discussion

MNP-Fe_3_O_4_ with various distinguished features attracts many attentions in biomedical fields, especially in tumor therapy. In this paper, an innovative technique has been developed by using MNP-Fe_3_O_4_ under ELMF exposure to generated localized physical cell damage and eventually kills tumor cells. Comprehensive studies have reported the biological effects of external static or alterable magnetic field in the last decades^31^,^32^,^33^,^34^,^35^,^36^,^37^,^38^,^39^. However, the specific mechanism of ELMF on biological system has not yet been fully investigated. According to the previous reports, it is suggested that ELMF with limited intensity (*e.g.* 0.3 μT at 10 Hz) could somehow cause a transient effect on cells, and result in recoverable cell damage^40^. In our experiment, similarly, we have also found that the exposure of cells to ELMF alone did not cause irreversible cell damage. On the other hand, it had been proved that ELMF could induce irreversible membrane pores formation due to the diamagnetic properties of cell membrane and the movement and vibration of MNPs could also be manipulated by external ELMF. In order to take full advantages of this phenomenon and consider the intriguing features of MNPs, we have combined the effects of MNPs and ELMF and explored their possibility in treating tumor cells.

To our interest, significant morphological changes can be observed in tumor cells treated with MNP-Fe_3_O_4_ under the exposure of ELMF, suggesting that loss of cell structure integrity induced by the combining effects of MNP-Fe_3_O_4_ and ELMF. To achieve effective cell killing, ELMF with various intensity (from 0.1 mT to 20 mT) and different frequencies (from 2 Hz to 20 Hz) was employed and evaluated for their efficiency in inducing irreversible tumor cell destruction, respectively. With the increase of intensity and frequency of ELMF stimulus, cell viability declined drastically, consistent with more significant morphological damages.

Most excitingly, in consistent with *in vitro* results, we have observed similar cell destruction in tumor tissues treated with MNPs and ELMF *in vivo*. Especially, under exposure of ELMF with appropriate intensity and frequency, tumor tissue injected with MNPs can be effectively destroyed with an obvious decrease of cell number and severe damage on their structural integrity.

To determine the specific pathway involved in the cell damage of the approach proposed in present study, it may require a thorough investigation regarding the mechanisms underlying the interaction between cells, MNPs and ELMF. Based on our results including cell morphology observation, cell viability evaluation, apoptotic cell staining, and *in vivo* animal analysis, we did observe significant structural changes in tumor cells upon treatment by MNPs and ELMF. Therefore, the possible mechanism for cell destruction is thus assumed that the movement and vibration o MNPs inside of cells under ELMF stimulus may aggravate the morphological changes of cells accompanying with apoptotic process. Future study will be conducted to pursue in-depth understanding in that regard.

In conclusion, we present a pilot study about utilizing MNPs in combination with ELMF stimulus to destruct tumor tissues by affecting cell structure. Combining effects of intracellular MNPs and its movement triggered by external ELMF result in significant morphological changes and finally contribute to effective cell killing. With the increase of the intensity and frequency of ELMF, more efficient cell damage can be achieved with the loss of structural integrity of tumor tissue.

## Materials and Methods

### Experimental setup

**Figure 7:**
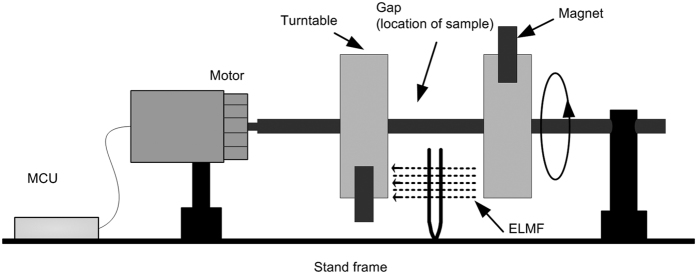
Schematic illustration of ELMF system.

Our in-house built ELMF system is designed and schematically described in Fig. 7. The entire system consists of a stand frame, two turntables, a motor, a microprogrammed control unit (MCU) and two permanent magnets. The permanent magnets are fixed on the turntables respectively and can provide a static magnetic field up to 50 mT. The two turntables together with magnets are connected with the motor, of which movement can be controlled by the MCU. All the components are placed and fixed on the stand frame. During ELMF treatment, the experimental samples were placed in the gap between two turntables, with a size of 20 mm in length, 15 mm in width and 30 mm in thickness. As the magnets can rotate at specific rate by controlling the movement of the motor connected with MCU, the frequency and intensity of magnetic field is thus tunable within the gap. In our experiment, the range of the magnetic field frequency is approximate 2–20 Hz and the range of magnetic field intensity is 0.1–20 mT.

### *In vitro* experiment

#### MNP-Fe_3_O_4_ characterization

MNP-Fe_3_O_4_ used in present study were coated with meso-2,3-dimercaptosuccinic acid (DMSA) to assure the stability of nanoparticles in biological solutions and also prevent its toxic effects. (Nanjing Nanoeast Biotech Limited Company, China). The morphology of particles was characterized using transmission electron microscopy (TEM). The particle size was measured using dynamic light scattering (DLS) system (Nano Partica SZ-100 series, Horiba Scientific Ltd., France).

#### Cell culture

MCF-7 cells (human breast adenocarcinoma cell line) were provided by China Pharmaceutical University, Nanjing, China. The cell culture media of Dulbecco’s modified Eagle medium (DMEM) was purchased from Gibco. MCF-7 cells were maintained as monolayer culture in DMEM medium supplemented with 10% fetal bovine serum (FBS) and 1% penicillin-streptomycin at 37 °C in a humidified atmosphere (5% CO_2_). When the cells reached at least 80% confluence, they were washed twice with phosphate-bufferedsaline (PBS) and detached with 0.25% trypsin/EDTA in Hank’s buffer (Invitrogen-GIBCO). An equal volume of medium with FBS for trypsin inactivation was then added and the tumor cells were collected, counted and used in different experiments.

#### Cytotoxicity test of MNPs

To investigate the cytotoxicity of MNPs, MCF-7 cells were seeded into 96-well plate at a density of 5 × 10^5^/ml and cultured for 24 h until adhesion. Then, the MNPs with concentration of [0, 5, 10, 15, 20] μg/ml in medium suspension were directly added into the each well, and the tumor cells were incubated with MNPs and continuously cultured for 24 h and evaluated for their viability with thiazolyl blue tetrazolium bromide (MTT) assay (ASTM E2526-08 standard method for estimating of cytotoxicity). For MTT assay, cultures after different treatments were incubated with cell culture medium containing MTT (0.5 mg/mL) for 3 hrs and then the reaction product of formazan crystal was extracted with dimethyl sulfoxide (DMSO). Absorbance of the extract was measured at 540 nm using a microplate spectrophotometer.

#### ELMF treatment

For ELMF treatment, MCF-7 cells (1 × 10^6^/ml) were seeded in cell culture petridishes (8 cm in diameter) and incubated at 37 °C (5% CO_2_) until attachment. MNPs (10 μg/ml) were then added and incubated with cells for 8 h allowing for sufficient cellular uptake. The cells were washed twice with PBS and dispersed into four small tubes with equal volume. Cells without MNPs but exposed with same ELMF were used as control.

Cells treated with/without MNPs were placed in the gap of ELMF system and exposed to various magnetic field, respectively. In each experiment, the cells were exposed at different frequency and intensity for 1 h. Cells in each group were exposed with ELMF at different magnetic field intensity (0.1, 5, 10, 15, 20 mT) and frequency (2, 5, 10, 15, 20 Hz), respectively. After 24 h, the morphological changes of cells in each experimental group were captured by an Olympus IX51 microscope. And the cell viability value quantified by MTT assay was plotted showing the trend of changes upon different treatments.

Cells were stained with apoptotic cell Hoechst 33258 dye kit to evaluate the damage effects. After various treatments, cells were first centrifuged and then suspended in PBS at a concentration of 1 × 10^6^/ml. Cells were then seeded into the cell culture dish and incubated with Hoechst 33258 (5 μl/ml) at 4 °C for 20 min. Afterwards, fluorescence images of cells in different groups were captured by using a LEICA TCS 4 D Confocal Microscope supplemented with a laser (400–500 nm).

### *In vivo* experiment

Female C57BL/6 mice (4 weeks, weighted 18–22 g), were purchased from Charles River Japan, Inc. To prepare tumor-bearing animals, the MCF-7 cell suspensions (1 × 10^6^/ml) with 0.1 ml PBS were injected subcutaneously into the subcutaneous tissue of C57BL/6 mice, which were anesthetized by intra-peritoneal injection of sodium pentobarbital (50 mg/kg of body weight). In our *in vivo* experiment, tumor diameters were measured every 3 days (starting from the day of implantation) by an external caliper, and the greatest longitudinal diameter (length) and the greatest transverse diameter (width) were determined. The tumor volume was calculated by the modified ellipsoidal formula: tumor volume = (largest diameter) × (smallest diameter)2 × 0.5^41^,^42^.

The mice were bred and maintained in specific pathogen-free conditions with a 12-h light/dark cycle and constant temperature of 24 °C in the animal facility at Nanjing University of Aeronautics and Astronautics (NUAA). All animal experiment methods were carried out in accordance with institutional guidelines of NUAA. All experimental protocols were approved by the Animal Committee of NUAA.

After subcutaneous nodules had grown to 5 mm in diameter, mice were then divided into three groups (8 mice in each group), MNPs (0.1 mg/g of body weight) was injected longitudinally into each nodule subcutaneously from the nodule edge. The mouse was under anesthesia when they were injected with MNPs. After maintained in specific pathogen-free conditions with constant temperature of 24 °C for 8 h, they were exposed to ELMF. During the treatment, tumor-bearing mice were placed in the gap area of ELMF system, exposed to the ELMF with frequency of 2 Hz and 20 Hz and intensity at 1 mT and 10 mT for 1 h, respectively. Mice injected intratumorally with the same volume of physiological saline and exposed in the ELMF were used as control group. After exposed to ELMF, the mice were maintained in specific pathogen-free conditions with constant temperature of 24 °C for 24 h, then they were sacrificed. The time is the same as the cell experiment.

The experiment results were observed according to the routine pathological section examinations. Briefly, the specimens of tumor tissue after various treatment were harvested, well-fixed in 10% buffered formalin, dehydrated in a graded ethanol series and then embedded in paraffin wax. Thin cross-sections (5 μm thick) were cut and stained with hematoxylin and eosin (H&E) to examine cell morphology under microscope.

### Statistics analysis

Each experiment was repeated at least three times, and the data are represented as mean ± standard error of the mean. Statistical analysis (unpaired t test) was performed using Origin software, version 8.0 (Origin Software, Massachusetts, CA). Differences were considered to be statistically significant when p < 0.05. For *in vivo* study, 8 mice per group were used for statistics analysis.

## Additional Information

**How to cite this article:** Li, W. *et al*. Evaluation of Tumor Treatment of Magnetic Nanoparticles Driven by Extremely Low Frequency Magnetic Field. *Sci. Rep.* **7**, 46287; doi: 10.1038/srep46287 (2017).

**Publisher's note:** Springer Nature remains neutral with regard to jurisdictional claims in published maps and institutional affiliations.
